# Anti-tumor effects of a recombinant anti-prostate specific membrane antigen immunotoxin against prostate cancer cells

**DOI:** 10.1186/s12894-017-0203-9

**Published:** 2017-02-13

**Authors:** Ping Meng, Qing-chuan Dong, Guang-guo Tan, Wei-hong Wen, He Wang, Geng Zhang, Yan-zhu Wang, Yu-ming Jing, Chen Wang, Wei-jun Qin, Jian-lin Yuan

**Affiliations:** 1 Department of Urology, Xijing Hospital, Fourth Military Medical University, Xi’an, Shaanxi China; 2 Department of Urology Surgery, Peoples’ Hospital of Shaanxi Province, Xi’an, Shaanxi China; 3 Department of Pharmaceutical Analysis, School of Pharmacy, Fourth Military Medical University, Xi’an, Shaanxi China; 4 Department of Immunology, Fourth Military Medical University, Xi’an, Shaanxi China; 5 Department of Urology, Tangdu Hospital, The Fourth Military Medical University, Xi’an, Shaanxi China; 6 State Key Laboratory of NBC Protection for Civilian, Beijing, China

**Keywords:** Gene therapy, Prostate cancer, Prostate-specific membrane antigen, Recombinant protein, Apoptosis

## Abstract

**Background:**

To evaluate anti-prostate cancer effects of a chimeric tumor-targeted killer protein.

**Methods:**

We established a novel fusion gene, immunocasp-3, composed of NH2-terminal leader sequence fused with an anti-prostate-specific membrane antigen (PSMA) antibody (J591), the furin cleavage sequences of diphtheria toxin (Fdt), and the reverse coding sequences of the large and small subunits of caspase-3 (revcaspase-3). The expressing level of the immunocasp-3 gene was evaluated by using the reverse transcription-PCR (RT-PCR) and western blot analysis. Cell viability assay and cytotoxicity assay were used to evaluate its anti-tumor effects in vitro. Apoptosis was confirmed by electron microscopy and Annexin V-FITC staining. The antitumor effects of immunocasp-3 were assessed in nude mice xenograft models containing PSMA-overexpressing LNCaP cells.

**Results:**

This study shows that the immunocasp-3 proteins selectively recognized and induced apoptotic death in PSMA-overexpressing LNCaP cells *in vitro*, where apoptotic cells were present in 15.3% of the cells transfected with the immunocasp-3 expression vector at 48 h after the transfection, in contrast to 5.5% in the control cells. Moreover, LNCaP cells were significantly killed under the condition of the co-culture of the immunocasp-3-secreting Jurkat cells and more than 50% of the LNCaP cells died when the two cell lines were co-cultured within 5 days. In addition, The expression of immunocasp-3 also significantly suppressed tumor growth and greatly prolonged the animal survival rate *in vivo*.

**Conclusion:**

A novel fusion gene, immunocasp-3, may represent a viable approach to treating PSMA-positive prostate cancer.

## Background

Prostate cancer is one of the most prevalent malignancy among males in Western countries [1]. Androgen ablation therapy are typically used to treat metastatic prostate cancer and advanced prostate cancer [2]. However, most of prostate cancer eventually develop to castration resistant prostate cancers(CRPC),then progressing rapidly [3]. Therefore, it is urgent to develop new molecule-targeted therapies.

Prostate-specific membrane antigen (PSMA) is a type II membrane glycoprotein of 100 kD, containing a transmembrane domain,a short intracellular segment and an extensive extracellular domain [4]. The expression of PSMA was independent of androgen, which was increased along with disease progression and reached to highest level in CRPC [5–7]. Moreover, the expression of PSMA is abundant in new vessels associated with the tumor but not in normal vessels [8–11]. Considering these points, the combination of gene therapy with anti-PSMA antibody represents an ideal treatment measure.

Caspase-3, one of critical cysteine protease family members, takes an important part in the signal transduction pathways that mediate apoptosis in mammalian cells [12]. Cleavage of immunotoxins was mediated by Furin, which was a rate-limiting step for several secreted proteins to produce cytotoxic activity [13]. Wang tao *et al*. described the construction and characterization of e23sFv-Fdt-revcaspase 3 containing the furin cleavage sequences (Diphtheria toxin, A_187_GNRVRRSVG_196_, Fdt) and concluded that the activity of immunocasp-3 proteins is comparable to that of PEA II-caspase 3, as they contain much less exogenous fragments [14].

**Fig. 1:**
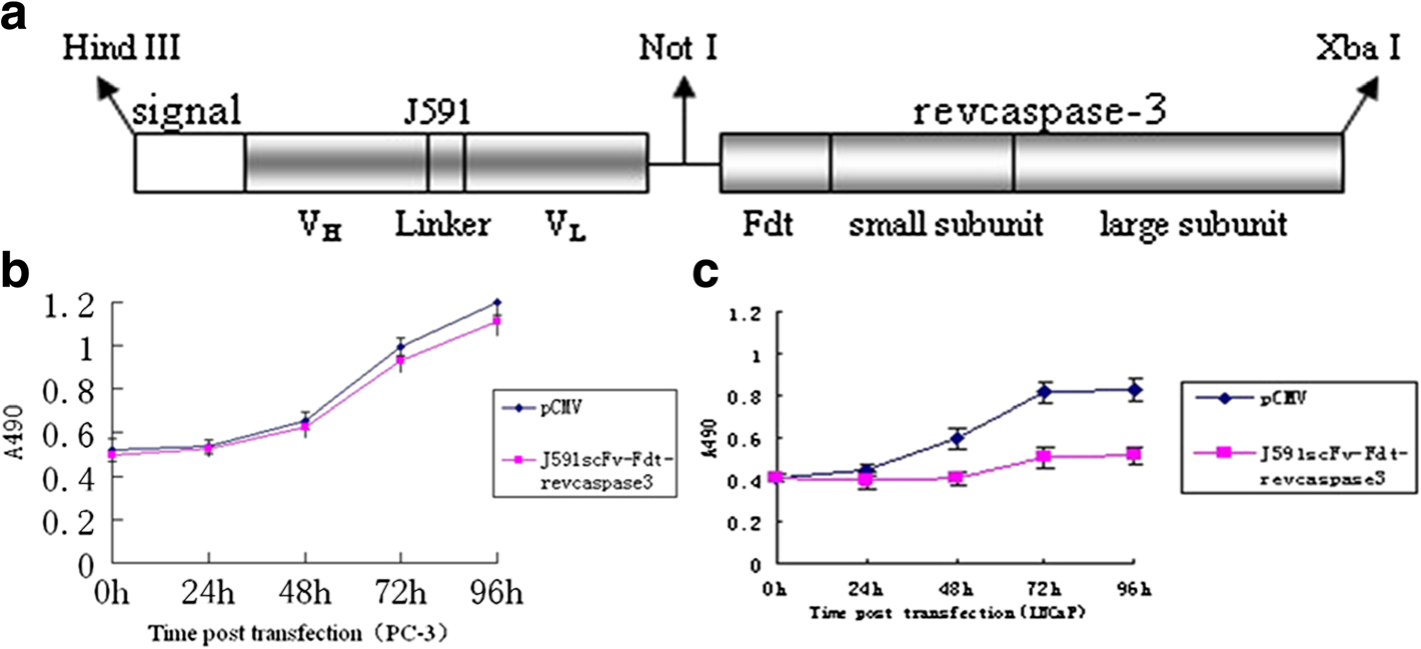
Expression of immunocasp-3 in PC-3 and LNCaP cells. **a**: Schematic diagram of immunocasp-3 comprising signal sequence, an anti-PSMA antibody (J591), the furin cleavage sequences of diphtheria toxin (Fdt), and reversed caspase-3 (revcaspase-3). MTT assay of PC-3 (**b**) and LNCaP cells (**c**) transfected with immunocasp-3

To develop a more effective and specific agent for antitumor therapy, we established a novel fusion gene, immunocasp-3 in this work, which was composed of NH2-terminal leader sequence fused with an anti-PSMA antibody (J591), the furin cleavage sequences of diphtheria toxin (Fdt), and the reverse coding sequences of the large and small subunits of caspase-3 (revcaspase-3) (Fig. 1a). The antitumor activities of immunocasp-3 and immunocasp-3 -secreting lymphocytes were evaluated *in vitro* and *in vivo*.

## Methods

### Cells lines

Two human prostate adenocarcinoma cell lines (LNCaP cells and PC-3 cells) and human Jurkat cells (American Type Culture Collection, Rockville, MD) were cultured in RPMI 1640 medium supplemented with 10% heat-inactivated fetal bovine serum. The response to PSMA for LNCaP and PC-3 cells was positive and negative, respectively, which has been confirmed in the previous study [15].

### Antibodies and plasmids

The hybridoma of J591 was purchased from the American Type Culture Collection (Rockville, MD). The plasmid pCMV-Fdt-revcaspase 3 was provided by Dr. Angang Yang (Fourth Military Medical University, Xi An, China).

### Mice

Four-to-six-week-old male nude mice, obtained from the Laboratory Animal Research Center of Fourth Military Medical University. All animal experiments were fully approved by the Administrative Committee of Experimental Animal Care and Use of Fourth Military Medical University, and conformed to the National Institute of Health guidelines on the ethical use of animals.

### Plasmids construction

A set of primers to amply the whole variant region sequences of heavy chain(VH) and light chain (VL) of murine antibodies were used to acquire VH and VL gene from hybridoma J591. HindIII, NotI site sequences, and a signal peptide sequence (MKHLWFFLLLVAAPRWVLS) were incorporated into J591 fragments by PCR. Fdt-revcaspase 3 was amplified by PCR using a pCMV-Fdt-revcaspase 3 plasmid as the template. The establishment of the recombinant genes was involved in the sequential fusion of the genes, which could encode J591, Fdt, and revcaspase 3. The recombinant genes were cloned downstream in the expression vector pCMV (Fig. 1a). The vector sequences were validated by DNA sequencing.

### Cell transfection

Twenty-four hours prior to transfection, LNCaP cells and PC-3 cells were seeded in 24-well plates at a density of 1 × 10^5^ cells per well. The transfection was performed by using Lipofectamine 2000 (Invitrogen, Carlsbad, CA) according to the standard procedure of the kit. the cells were selected in the medium consisting of 800 μg/ml G418 (Invitrogen, Carlsbad, CA) for two to three weeks. The cells were cultured in the medium consisting of 800 μg/ml G418 (Invitrogen, Carlsbad, CA) for two more weeks to select stable transfection.

### Cell viability assay

The viability of the cells was assessed using the 3-(4,5-dimethylthiazol-2-yl)-2,5-diphenyltetrazolium bromide (MTT) reduction assay. In the MTT assay, the yellow tetrazolium salt (MTT) is reduced in metabolically active cells to form insoluble purple formazan crystals, which are solubilized by the addition of a detergent. The color can then be quantitated by spectrophotometry. The cells transfected with the immunocasp-3 gene were cultured in 96-well plates for 24 to 96 h. Cells were then incubated with 20 μL of MTT (1.5 mg/mL; Sigma-Aldrich) per well for 4 h at 37 °C. Cells were centrifuged at 800 rpm for 10 min, and then 150 μL of DMSO was added and mixed by gentle pipetting to solubilize the cells. The optical density of the solution was read at 490 nm using a Universal Microplate Reader (Bio-Tek Instruments, Inc.).

### Western blot analysis

We separated The lysates of transfected cells and the serum-free supernatant fluids of cells transfected with immunocasp-3 permanently by SDS-PAGE. Then proteins of cells were blotted onto polyvinylidene difluoride membranes (Amersham Pharmacia Biotech), and then we incubated these membranes with primary antibodies which recognize caspase-3 (1:500; BD PharMingen) at 4 °C in PBST overnight. Next, we washed out the primary antibodies and changed to horseradish peroxidase-conjugated secondary antibody (1:2,000; ZhongShan), incubating for 2 h at room temperature. Immunoreactive bands were detected by chemiluminescence kit (Pierce).

### Electron microscopy

Pellets of cells were fixed with with 2.5% glutaraldehyde in 0.1 mol/L sodium phosphate buffer (pH 7.4) for 2 h at 4 °C. Then after being washed 3 times, and they were fixed at second time in 1% osmic acid in phosphate buffer before scrapping, dehydration, and embedding. Ultrathin sections mounted on 200 mesh grid were examined in a GEM-2000EX electron microscope.

### Cytotoxicity assay in vitro

Transwell filters (Costar), (diameter: 12 mm, pore size: 0.40 μm), which separate the cells but not large molecules, were used for the cocultivation assay. Tumor cells overexpressing PSMA (LNCaP) and control cells expressing undetectable PSMA (PC-3) were placed at the bottom chamber, and the transfected Jurkat cells were placed at the top chamber. Viable cells at the bottom chambers were numbered by trypan blue exclusion at the indicated time points after cocultivation. The percentages of cell killing were calculated as follows: 1 - (the number of cells cocultured with stably transfected Jurkat/the number of cells cocultured with Jurkat controls) × 100%.

### Antitumor activity of immunocasp-3 in vivo

Four-to-six-week-old male nude mice were inoculated with 2 × 10^6^ human prostate cancer LNCaP cells by administering a subcutaneous injection of the cells in the right hind flank. Tumors were allowed to grow until they reached a diameter of 5–7 mm (day 0). The mice were then randomly divided into different treatment groups of 5 mice each. One group of mice received 6 doses (twice a week) of 10 μg of pCMV-J591-Fdt-revcaspase 3 mixed with lipofectamine as intramuscular injections administered in the right posterior limb. Another group of 5 mice received 3 weekly intravenous injections of 2 × 10^6^ Jurkat-J591-Fdt-revcaspase 3 cells. The control group was injected with either liposome-mixed empty pCMV vector or unmodified Jurkat cells. Tumor growth was monitored with measurement of 2 perpendicular tumor diameters every 3 days using a caliper, and the volume of the tumor in mice was calculated by formula: tumor volume = (width)^2^ × length/2. The survival time of the mice was assessed. The mice were then sacrificed by cervical dislocation and dissected, tissues were washed and fixed in 10% neutral-buffered formalin, and then embedded in paraffin. These paraffin-embedded tissue sections were dewaxed, hydrated, incubated in 0.3% methanol-H_2_O_2_ for 20 min to remove endogenous peroxidase and then stained with rabbit anti-human active caspase-3, as described above.

### Statistical analysis

Statistical analysis was performed using the SPSS12.0 software package for Windows (SPSS). Survival rates were analyzed by the Kaplan-Meier method, and intergroup comparisons were made using the log-rank test. Statistical significance was defined as P≦0.05.

## Results

### Immunocasp-3 fusion proteins expressed in PC-3 and Jurkat cells

PC-3 cells were transiently transfected with the immunocasp-3 expression vector or the pCMV void vector. Translocation was evaluated indirectly by measuring the degree of caspase-3-induced cytotoxicity. The proliferation properties of immunocasp-3-expressing PC-3 cells were similar to those of cells transfected with the pCMV void vector, which suggests that the expression of the immunocasp-3 fusion protein was not toxic (Fig. 1b).

**Fig. 2:**
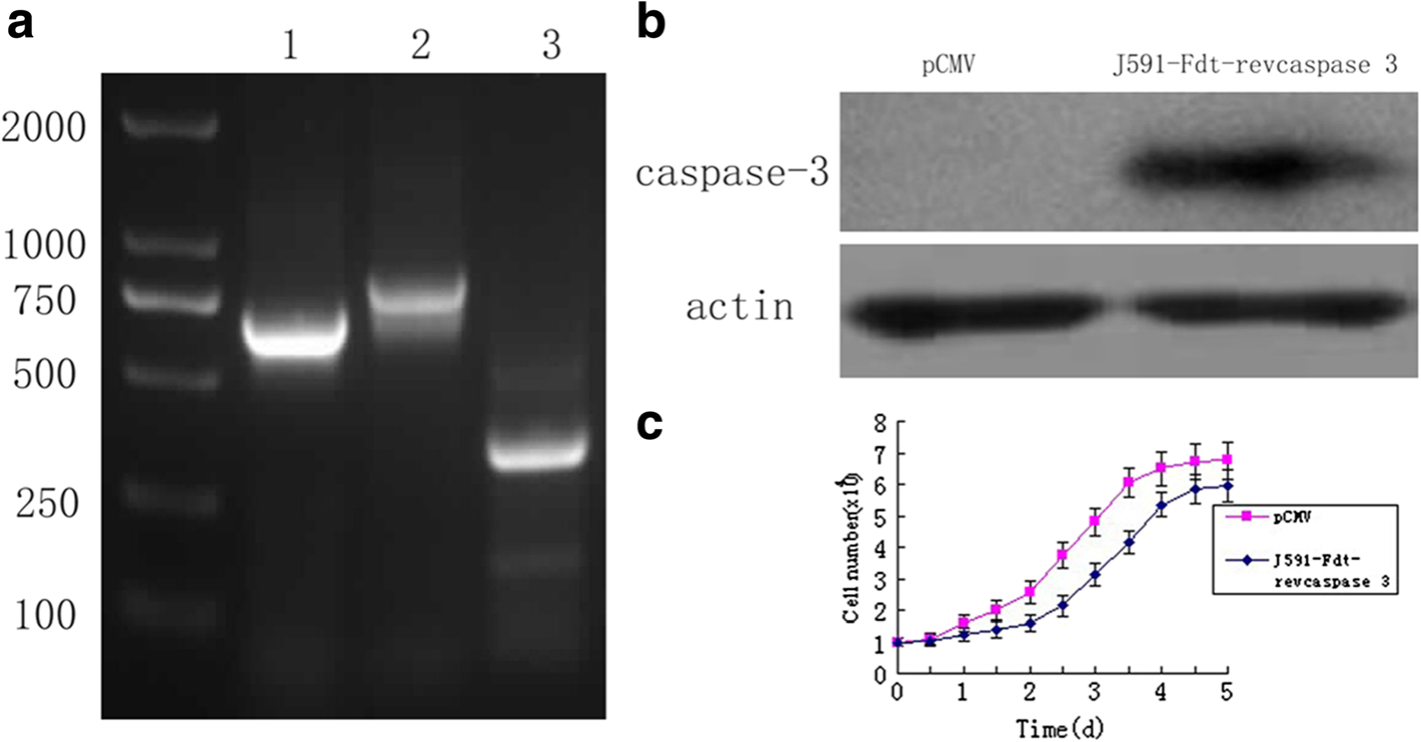
Detection of immunocasp-3 protein secreted by genetically modified Jurkat cells. **a**: Genomic DNA was isolated from the immunocasp-3-modified Jurkat cell clones and analyzed by PCR: J591 (lane 1); revcaspase-3 (lane 2); β-actin (lane 3), respectively. **b**: Western blot analysis of the concentrated cell culture medium obtained from Jurkat cells stably transfected with the immunocasp-3 gene. Blots were probed with the anti-caspase-3 antibody. **c**: Growth and proliferation characteristics of Jurkat cells transfected with immunocasp-3 or unmodified Jurkat cells

CD4^+^ Jurkat cells were then transfected with the immunocasp-3 expression vector and stable-expression cells were selected by the G418 selection method; the expression of the vector was expected to induce these cells to secrete the targeted protein, which in turn would kill PSMA-overexpressing tumor cells. The expression of the immunocasp-3 gene was detected by reverse transcription-PCR (RT-PCR) with primers specific to anti-PSMA antibody fragment (J591) and Fdt-revcaspase 3 (Fig. 2a). The expression and secretion of fusion proteins were confirmed by western blot for cell culture supernatants (Fig. 2b). Consequently, the immunocasp-3-modified Jurkat cells remained alive and showed similar growth and proliferation properties to those of unmodified cells (Fig. 2c), suggesting that the expression of the chimeric protein is associated with low toxicity.

### Immunocasp-3 fusion proteins specifically kill PSMA-overexpressing tumor cells in vitro

**Fig. 3:**
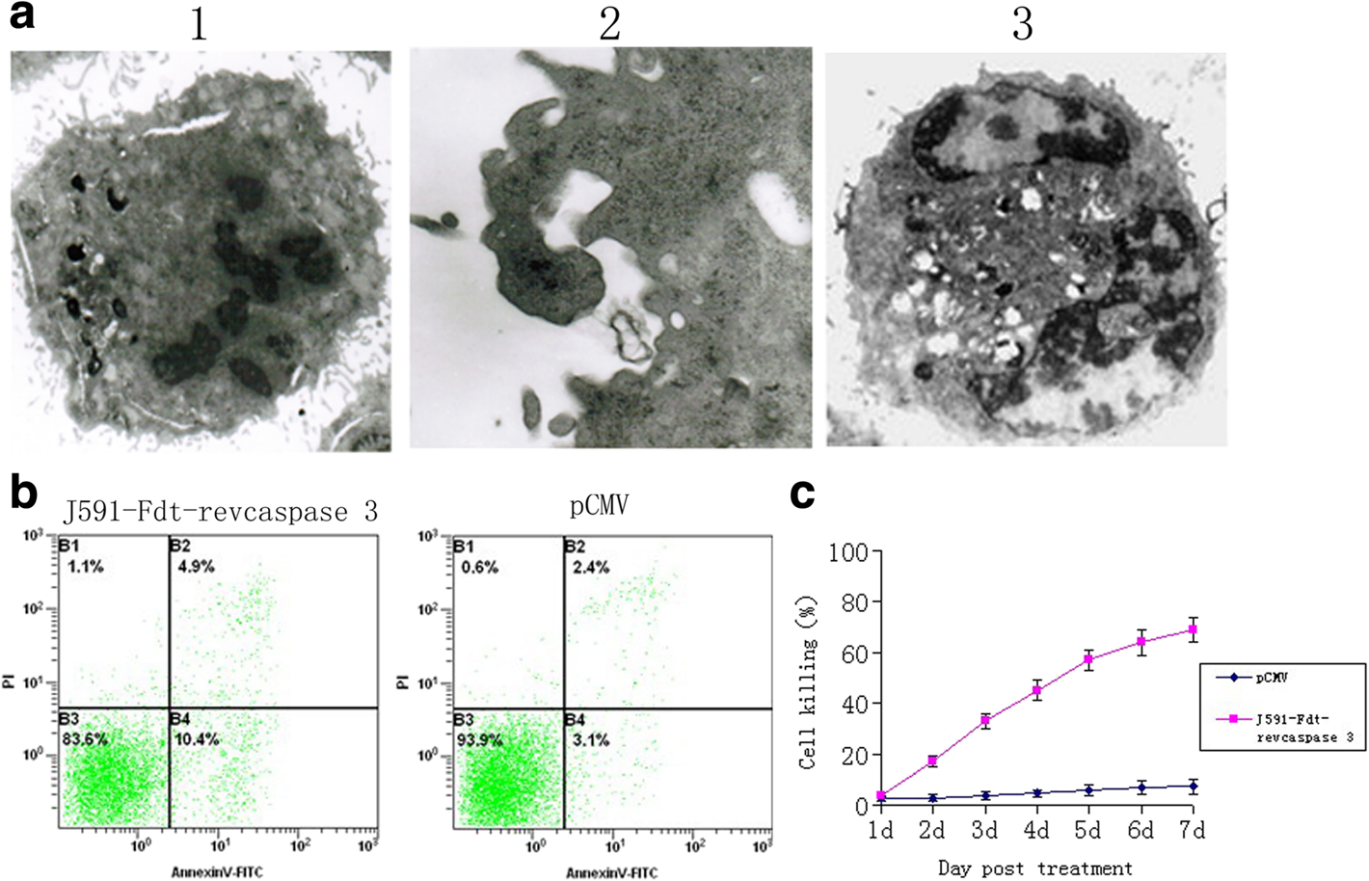
Immunocasp-3 fusion proteins inhibit the growth of LNCaP cells. **a**: Electronic microscopy of LNCaP 48 h after transfection of immunocasp-3. **b**: 48 h after transfection, LNCaP cells were subjected to Annexin V-FITC staining concomitant with 4,6-diamidino-2-phenylindole nucleus staining and analyzed by flow cytometry. **c**: LNCaP cells were cocultured with immunocasp-3-expressing Jurkat cells for the indicated time points, and the percentages of killing were calculated

Fusion proteins were transiently expressed in PSMA-positive LNCaP and PSMA-negative PC-3 cells. Cell death was observed apparently in LNCaP (Fig. 1c) cells but not in PC-3 cells at 48 h after transfection. Cytotoxicity was not due to the immunocasp-3 secretion, but rather the result of PSMA expression on the surface of LNCaP cells. Typical apoptotic changes were observed in cells expressing immunocasp-3 by the electronic microscope, including chromatin condensation and margination at the nuclear periphery, cellular shrinkage and blebbing, and formation of apoptotic bodies (Fig. 3a). Moreover, using FITC-annexin V staining of the cells, it was revealed that apoptotic cells were present in 15.3% of the cells transfected with the immunocasp-3 expression vector at 48 h after the transfection (Fig. 3b), in contrast to 5.5% in the control cells, a percentage which was higher than that in the control cells. The genetically modified Jurkat cells were then cocultivated in vitro with PSMA-positive LNCaP and PSMA-negative PC-3 cells. The ratio of Jurkat: target cells was adjusted to 3:1, basing on pilot experiments of optimal ratio for cell killing. As shown in Fig. 3c, a significant number of LNCaP cells, but not PC-3 cells, were killed by immunocasp-3-secreting Jurkat cells. More than 50% of the cells had died within 5 days of coculture, and additional killing of PSMA-overexpressing tumor cells was seen after longer times.

Taken together, we conclude that immunocasp-3 proteins are capable of recognizing and killing PSMA-positive LNCaP cells, but not PSMA-negative PC-3 cells in vitro.

### Immunocasp-3 fusion proteins effectively suppress the growth of PSMA-overexpressing tumor cells in vivo and prolongs survival in nude mice

**Fig. 4:**
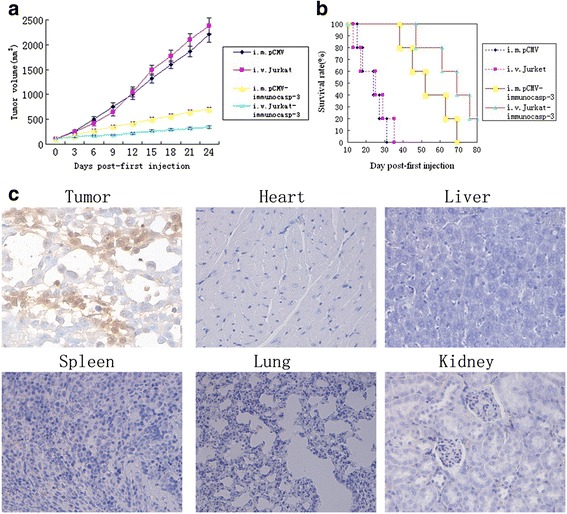
The antitumor activity of immunocasp-3 on PSMA-overexpressing tumors in vivo. **a**: Tumor volume and tumor growth curves in mice injected with lipofectamine-encapsulated immunocasp-3 gene or pCMV plasmid or with immunocasp-3 gene-modified Jurkat cells or control Jurkat cells. **b**: Survival of mice after treatment, as described in **a. c**: Nude mice with LNCaP tumor received 3 weekly intravenous. injections of 2 × 10^6^ immunocasp-3 gene-modified Jurkat cells. Tissues were then subjected to immunohistochemical analysis with an anti-caspase-3 antibody

The in vivo antitumor activity of immunocasp-3 in nude mice xenograft models containing PSMA-overexpressing LNCaP cells were assessed by measuring the tumor size and animal survival rate. LNCaP cells were subcutaneously inoculated into nude mice to form solid tumors. Murine xenograft models were randomly divided into two treatment groups. One group of mice were received 6 doses of 10 μg of pCMV-J591-Fdt-revcaspase 3, or empty pCMV every 3 days over the course of the study. Another group of prostate cancer-bearing mice received 3 weekly intravenous. injections of 2 × 10^6^ Jurkat cells expressing J591-Fdt-revcaspase-3 or unmodified Jurkat cells. Both the vector-lipofectamine-treated group and Jurkat-cell-treated groups showed greater decrease in tumor volume and longer survival time than controls (Fig. 4a and b). Meanwhile, Jurkat-cell-treated group was more efficient than the vector-lipofectamine group in reducing tumor size (*P* < 0.05), suggesting that this strategy of gene administration results in more effective and stable antitumor activities in LNCaP xenografts. Immunohistochemical analysis also confirmed the presence of caspase-3 activity in tumors treated with Jurkat cells expressing J591-Fdt-revcaspase 3, but not in the normal tissues (Fig. 4c).

## Discussion

Many strategies have been designed to kill cancer cells [16–18]. Because of its restricted and abundant surface expression on prostate cancer cells, PSMA constitutes an attractive target for immunotherapies against prostate cancer. Many papers have already reported that the combination of gene therapy and anti-PSMA antibody receives an ideal therapeutic result [19–24]. Indeed, the antibody used in this study, J591, has been previously used for the treatment of prostate cancer cells [23, 25–29].

Wild-type caspase-3 consists of an NH2-terminal prodomain, a large subunit, and a small subunit. However, active caspase-3, which is constructed with the reverse order of the subunits [30], can induce apoptosis of tumor cells without apoptotic signals; these properties make active caspase-3 an attractive candidate molecule for gene therapy. In this study, the caspase 3 gene was generated by reversing the order of the coding sequences for the large and small subunits [31]. To achieve successful proapoptotic gene therapy for prostate cancer cells, it is necessary to devise a gene construct that expresses the proapoptotic gene selectively in the prostate cells. Therefore, we generated a novel immunocasp-3 gene by fusing a leader sequence, an anti-PSMA antibody (J591) and the furin cleavage sequences of diphtheria toxin (Fdt) to the active revcaspase-3 [14]. The results of our in vitro and in vivo studies revealed that the resultant protein, immunocasp-3, killed PSMA-overexpressing tumor cells, but not PSMA-negative cells. PC-3 cells are PSMA-negative tumor cells. Immunocasp-3 fusion proteins secreted in the culture media cannot enter PC-3 cells via receptor-mediated endocytosis, and thus cause no damage. Our results showed that PC-3 cells expressing recombinant Immunocasp-3 fusion proteins proliferate normally. Once internalized by PSMA-overexpressing tumor cells such as LNCaP cells, immunocasp-3 proteins are exposed to a low-pH environment in the endosome, where the peptide bond in the Fdt domain is cleaved by furin. Then immunocasp-3 proteins release COOH-terminal fragments, which consequently translocate to the cytosol and induce PSMA-overexpressing tumor cells to apoptosis. Both injection of lipofectamine-encapsulated immunocasp-3 and infusion of immunocasp-3 gene-modified Jurkat cells could suppress tumors due to continuous secretion of the killer protein and its diffusion through lymph fluid and blood. However, compared with direct injections of lipofectamine-encapsulated immunocasp-3, the applyment of immunocasp-3 gene-modified Jurkat cells may be a more effective and convenient therapeutic method, which simplified the procedure like protein purification. Moreover, caspase-3 proteins were endogenous human proteins that not only kill prostate cancer cells in a physiological manner, but also resulted in relatively weak immunogenicity and minor general toxicity over repeated administrations. However, because of immunogenicity of murine antibodies, fully human antibodies could become necessary.

## Conclusions

The newly immunocasp-3 is both highly specific and effective against PSMA-overexpressing prostate cancer cells. Treatment using this gene merits further investigation and consideration as a molecularly targeted therapeutic measure for prostate cancers.

## Abbreviations

MTT: 3-(4,5-dimethylthiazol-2-yl)-2,5-diphenyltetrazolium bromide
PSMA: Anti-prostate-specific membrane antigen
SPSS: Statistical software package for social sciences
VH: Heavy chain
VL: Light chain
